# MNF, an ankyrin repeat protein of myxoma virus, is part of a native cellular SCF complex during viral infection

**DOI:** 10.1186/1743-422X-7-56

**Published:** 2010-03-08

**Authors:** Sophie Blanié, Jacqueline Gelfi, Stéphane Bertagnoli, Christelle Camus-Bouclainville

**Affiliations:** 1 INRA, UMR 1225 Interactions Hôtes - Agents Pathogènes F-31076 Toulouse, France; 2 Université de Toulouse; ENVT; UMR 1225 Interactions Hôtes - Agents Pathogènes; F-31076 Toulouse, France

## Abstract

Myxoma virus (MYXV), a member of the *Poxviridae *family, is the agent responsible for myxomatosis, a fatal disease in the European rabbit (*Oryctolagus cuniculus*). Like all poxviruses, MYXV is known for encoding multiple proteins that regulate cellular signaling pathways. Among them, four proteins share the same ANK/PRANC structure: M148R, M149R, MNF (Myxoma Nuclear factor) and M-T5, all of them described as virulence factors. This family of poxvirus proteins, recently identified, has drawn considerable attention for its potential role in modulating the host ubiquitin-proteasome system during viral infection. To date, many members of this novel protein family have been shown to interact with SCF components, *in vitro*. Here, we focus on MNF gene, which has been shown to express a nuclear protein presenting nine ANK repeats, one of which has been identified as a nuclear localization signal. In transfection, MNF has been shown to colocalise with the transcription factor NF-κB in the nucleus of TNFα-stimulated cells. Functionally, MNF is a critical virulence factor since its deletion generates an almost apathogenic virus. In this study, to pursue the investigation of proteins interacting with MNF and of its mechanism of action, we engineered a recombinant MYXV expressing a GFP-linked MNF under the control of MNF native promoter. Infection of rabbits with MYXV-GFPMNF recombinant virus provided the evidence that the GFP fusion does not disturb the main function of MNF. Hence, cells were infected with MYXV-GFPMNF and immunoprecipitation of the GFPMNF fusion protein was performed to identify MNF's partners. For the first time, endogenous components of SCF (Cullin-1 and Skp1) were co-precipitated with an ANK myxoma virus protein, expressed in an infectious context, and without over-expression of any protein.

## Findings

Myxoma virus (MYXV), a member of the *Poxviridae *family, is the agent responsible for myxomatosis, a fatal disease in the European rabbit (*Oryctolagus cuniculus*). Due to its host specificity, MYXV is a useful model to study *in vivo *the mechanisms by which the numerous virus-encoded virulence factors control host response to infection. Among the virulence factors, MYXV genome encodes four proteins which share the same structure: M148R, M149R, MNF and M-T5 [1-4]. They present Ankyrin repeats (ANK) and a C-terminal PRANC domain [5]. The Ankyrin repeat, a 33-residue sequence domain, is one of the most common protein-protein interaction motifs found in nature [6]. Extensively identified in eukaryotes and bacteria, ANK motif is relatively rare among viruses, with the exception of poxviruses, especially chordopoxviruses [7]. Thus, poxviruses generally encode 4 or 5 ANK proteins, and more than 80% of these ANK proteins present an F-box-like domain at their C-terminus [5]. This domain is shorter than the typical cellular F-box and lacks helix 3 [8]. Furthermore, the poxviral F-box-like domain is located at the C-terminus of ANK protein whereas cellular F-boxes are typically located in the N-terminus of the protein. In addition, cellular F-boxes are associated with a second protein-protein interaction domain such as WD40 or Leucine Rich Repeat but no association with ANK motif has been reported yet. Due to these significant differences with eukaryotic F-boxes, the poxviral F-box domain has been defined as a new motif: the PRANC domain (Pox protein Repeats of Ankyrin C-terminal) [9].

The F-box motif mediates protein-protein interactions and was first described as a recognition motif in the E3 ubiquitin ligase complex, known as SCF (Skp, Cullin, F-box) [10]. The SCF complex is a multi-protein complex that mediates the ubiquitination of substrates mainly destined for degradation by the 26S proteasome [11]. Targeted proteins are recognized by a variety of F-box proteins and are delivered to the E3 ligase complex via an adaptor protein, Skp1 [12]. Interaction between the adaptor protein and the F-box protein occurs via the F-box motif. The SCF complex plays a critical role in the selective degradation of regulatory proteins that mediate various cellular functions, such as signal transduction and cell cycle regulation [12]. Ten poxvirus ANK proteins have been shown to contain a functional F-box: myxoma virus protein M-T5 [13], orf virus proteins OV008, OV123, OV126, OV128 and OV129 [9], vaccinia virus protein MVA186R [14] and ectromelia virus proteins ECTV002, ECTV005 and ECTV154 [15]. Hence, among MYXV proteins, only M-T5 was identified as a cellular binding partner of Cullin-1 to date. Enhanced ubiquitination of p27/Kip1 and subsequent degradation via the proteasome pathway was observed through the interaction of M-T5 and Cullin-1. Consistent with this interaction, M-T5 was shown to promote cell cycle progression beyond the G0/G1 checkpoint during virus infection. In this study, we focused on MNF. MNF gene expresses a nuclear protein that presents nine ANK repeats, one of which exhibits significant sequence similarity with IκBα nuclear localization signal. Moreover, in transfection, MNF colocalises with the transcription factor NF-κB in the nucleus of TNFα-stimulated cells. Functionally, infection of rabbits with an MNF-deleted MYXV induced very mild clinical signs, with few discrete secondary myxomas, no respiratory infection, and no lethality. Histological analysis of the primary myxoma and parotid lymph node showed that deletion of MNF gene allowed a more rapid and quickly resolved inflammation [3]. To summarize, MNF was shown to inhibit the pro-inflammatory pathway, seemingly through an interaction with NF-κB pathway.

**Figure 1:**
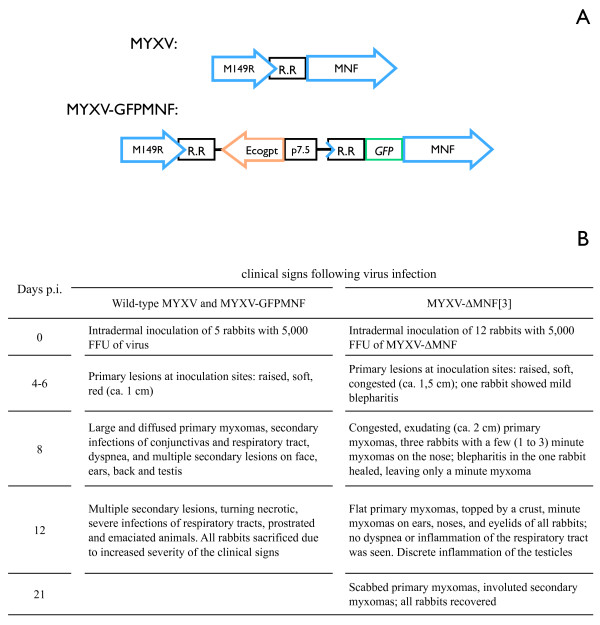
**Schematic representation of MNF gene region of wild-type MYXV, recombinant MYXV-GFPMNF and clinical signs associated**. A. Mutant virus was obtained by homologous recombination between wild type MYXV and a transfer plasmid. The resulting recombinant MYXV-GFPMNF encodes GFPMNF fusion, under control of MNF promoter. R.R. encompasses the end of M149R-coding sequence and the intergenic sequence between M149R and MNF. To select recombinant virus, the *Ecogpt *gene, under control of P7.5 early poxviral promoter, was inserted between M149R and GFPMNF. So as not to disturb expression of M149R or MNF by the selection gene insertion, R.R. was duplicated. B. Pathogenicity of wild-type and mutant viruses in European rabbits. Eight-week-old New Zealand White rabbits were obtained from a local supplier and housed in biocontainment facilities according to the guidelines of the European Community Council on Animal Care (European Council directive 86/609/EEC, 24 November 1986). All procedures on animals were performed by staff accredited by the French Ministry of Agriculture and were designed to limit animal pain and distress. Infections were performed intradermally in the right ear with 5 × 10^3 ^FFU of either virus. Rabbits were monitored daily for clinical signs of myxomatosis. Rabbits that became moribund were sacrificed with T61 administered intravenously.

**Figure 2:**
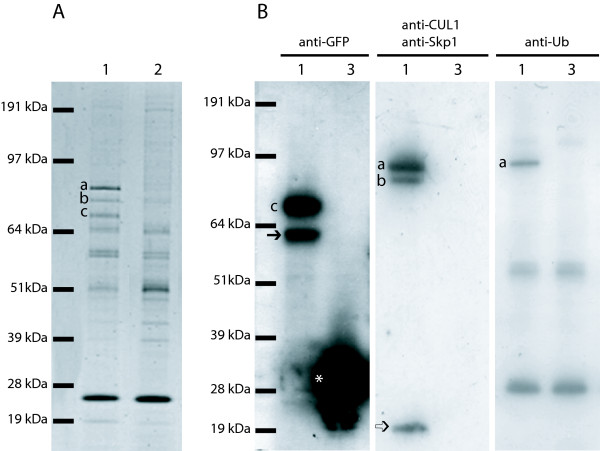
**Analysis of MNF partners by co-immunoprecipitation**. Ten mm plates of BGMK cells (Baby green monkey kidney) were infected with MYXV-GFPMNF (1), wild-type MYXV (2) or MYXV-GFP (3), at m.o.i 3. The latter has been obtained by insertion of GFP gene, under control of the P7.5 poxviral promoter, in the thymidine kinase locus. 24 hours post-infection, cells were washed in PBS and lysed with hypotonic buffer (10 mM HEPES pH7.9, 150 mM NaCl, 600 mM KCl, 0.5% NP40 and proteases inhibitors). Lysates were cleared by centrifugation and the supernatants incubated with μMACS anti-GFP MicroBeads. Washes and elution were performed according to manufacturer's instructions. Eluates were loaded on SDS/PAGE gels and analyzed by Simply Blue SafeStain (A) or by western blot (B). A. The 3 bands specific of MYXV-GFPMNF precipitation (a b and c) were analyzed by mass spectrometry. B. Western blot analysis of lysates with rabbit anti-GFP, rabbit anti-Cullin-1, rabbit anti-Skp1 or mouse anti-ubiquitin antibodies. Secondary antibodies were from anti-rabbit or anti-mouse WesternBreeze Chemiluminescent Kit, respectively. c and →: GFPMNF, *: GFP, a and b: Cullin-1 bands, and ⇒: Skp 1 band.

To go further in the identification of the proteins interacting with MNF in a viral infectious context, different assays to produce antibodies directed against MNF were performed. All the rabbit sera obtained by immunization against part of or the whole MNF protein were specific to the MYXV infection but allowed the staining of cells infected with the MYXVΔMNF too, indicating that they were not MNF-specific (data not shown). Thus, to overcome the difficulty to trace MNF by use of antibodies, a recombinant MYXV expressing a GFP-linked MNF, where GFP is in N-terminus of MNF, was engineered (MYXV-GFPMNF) (Figure 1A). The fusion protein GFPMNF was expressed under control of MNF native promoter. The *Ecogpt *selection gene (*Escherichia coli *xanthine-guanine phosphoribosyl transferase), under the control of the P7.5 early poxviral promoter, was inserted between M149R and GFPMNF. The regulating region (R.R.) between M149R and MNF might contain sequences necessary to M149R expression but also contains MNF promoter which begins at the end of M149R-coding sequence. So as not to disturb the regulating region, and thus the expression of M149R, by the insertion of the selection gene, the R.R. was duplicated. Recombinant virus was selected upon gpt selection and purity was verified by PCR (data not shown). The correct expression of the fusion protein, its impact on MNF functionality, and thus on MYXV pathogenicity, were verified. The purified wild-type, MYXV-GFPMNF and MYXVΔMNF [3] viruses were inoculated intradermally (5 × 10^3 ^Foci Forming Unit (FFU)) to the right ear of European rabbits (five animals per virus). Rabbits were monitored daily for clinical signs of myxomatosis. Rabbits infected with wild-type or MYXV-GFPMNF viruses developed a classic form of myxomatosis, totally different from the one induced by MYXV-ΔMNF virus which generated very mild clinical signs (Figure 1B). This experiment suggests that the main function of MNF is preserved even when MNF is fused to GFP. To identify the partners of MNF, immunoprecipitations of GFPMNF were performed. BGMK cells (Baby green monkey kidney) were infected with wild-type MYXV, MYXV-GFPMNF, MYXV-GFP or mock infected. Twenty-four hours post-infection (h.p.i.), cells were washed and lysed with hypotonic buffer. Lysates were cleared by centrifugation and the supernatants incubated with anti-GFP coupled to magnetic beads in order to precipitate GFPMNF fusion protein. Eluates were loaded on SDS/PAGE gels and analyzed by Coomassie blue staining. Three bands (Figure 2A a, b and 2A c) were clearly specific of MYXV-GFPMNF infection, and were thus excised, digested and analyzed by mass spectrometry. One of them (band c) was identified as the GFPMNF fusion protein and the two others as Cullin-1 (a and b). These data were confirmed by western-blot analysis of the lysates (Figure 2B left and middle panels). The presence of two GFPMNF bands suggests that during infection, MNF may be expressed under two forms, one being modified post-translation. Since the interaction between F-box protein and Cullin-1 usually occurs via the Skp1 protein, a western-blot analysis of the eluates was performed using anti-Skp1 antibodies (Figure 2B, middle panel). This experiment shows that Skp1 co-precipitates with MNF and suggests that Skp1 allows the interaction between MNF and Cullin-1. To identify a potential ubiquitinated substrate, an anti-ubiquitin western-blot was performed onto eluates (Figure 2B right panel). An ubiquitinated protein corresponding to band a (figure 2A), previously identified as Cullin-1 by mass spectrometry, was specifically co-precipitated with GFPMNF. This explains the two Cullin-1 bands identified by mass spectrometry, suggesting that the main part of MNF-co-precipitating Cullin-1 seems to be ubiquitinated. Cullin-1 auto-ubiquitination has already been reported [16] but its meaning remains to be clarified. However, in this experiment, no ubiquitinated substrate was co-precipitated with the SCF complex containing MNF. Since MNF is suspected to interact with NFκB pathway and IκB degradation occurs via its ubiquitination by SCF, variations in IκB expression were measured in cells infected with MYXV or MYXVΔMNF at various times post-infection. No differences were shown, suggesting that MNF does not interfere with degradation of IκB (Data not shown).

For the first time, endogenous components of SCF (Cullin-1 and Skp1) were co-precipitated with an ANK myxoma virus protein, virally expressed under control of its own promoter, and without over-expression of any protein. Thus, our data demonstrate that MNF interacts with cellular SCF ubiquitin ligase complexes, in real infectious context. Many viral proteins have been shown to interact with subunits of the SCF complex. In the case of ANK poxviral proteins, they seem to act as substrate adaptors, thus directing the ubiquitin ligase complex toward new targets or protecting cellular targets from ubiquitination. However, the substrates are mostly unknown. In some instances, viral proteins can also be targeted by SCF for their own destruction. The only identified substrates ubiquitinated by SCF containing an ANK/PRANC poxviral protein are p27 [13] and Akt [17], which are bound to the host SCF complex via Skp1 and MYXV M-T5 in infected cells. On the other hand, it has been shown that the 68k-ank protein contains another critical domain that may function independently from SCF ubiquitin ligase complex formation [18], suggesting that ANK/PRANC poxviral proteins may have multiple roles. In addition, poxviruses encode ANK proteins that do not present PRANC motif, such as Vaccinia virus K1L which mediates host-range function in RK-13 cells via ANK repeat. Moreover, K1L may interact with a cellular GTPase-activating protein [19] and inhibits host NFκB activation by preventing IκBα degradation [20]. The identification of proteins bound by the N-terminal ANK domains and the outcome of these proteins could also greatly enhance our understanding of the multiple functions of the ANK/PRANC poxviral proteins during infection.

## Competing interests

The authors declare that they have no competing interests.

## Authors' contributions

SBl carried out all the experiments and drafted the manuscript. CC, SBe and JG participated in the design of the study and revision of the manuscript. All authors read and approved the final manuscript.

## References

[B1] Cameron C Hota-Mitchell S Chen L Barrett J Cao JX Macaulay C Willer D Evans D McFadden G The complete DNA sequence of myxoma virus Virology 1999 264 298 318 10.1006/viro.1999.0001 10562494

[B2] Blanie S Mortier J Delverdier M Bertagnoli S Camus-Bouclainville C M148R and M149R are two virulence factors for myxoma virus pathogenesis in the European rabbit Vet Res 2009 40 11 10.1051/vetres:2008049 19019281 PMC2695013

[B3] Camus-Bouclainville C Fiette L Bouchiha S Pignolet B Counor D Filipe C Gelfi J Messud-Petit F A virulence factor of myxoma virus colocalizes with NF-kappaB in the nucleus and interferes with inflammation J Virol 2004 78 2510 2516 10.1128/JVI.78.5.2510-2516.2004 14963153 PMC369233

[B4] Mossman K Lee SF Barry M Boshkov L McFadden G Disruption of M-T5, a novel myxoma virus gene member of poxvirus host range superfamily, results in dramatic attenuation of myxomatosis in infected European rabbits J Virol 1996 70 4394 4410 8676463 10.1128/jvi.70.7.4394-4410.1996 PMC190373

[B5] Mercer AA Fleming SB Ueda N F-box-like domains are present in most poxvirus ankyrin repeat proteins Virus Genes 2005 31 127 133 10.1007/s11262-005-1784-z 16025237

[B6] Mosavi LK Cammett TJ Desrosiers DC Peng ZY The ankyrin repeat as molecular architecture for protein recognition Protein Sci 2004 13 1435 1448 10.1110/ps.03554604 15152081 PMC2279977

[B7] Bork P Hundreds of ankyrin-like repeats in functionally diverse proteins: mobile modules that cross phyla horizontally? Proteins 1993 17 363 374 10.1002/prot.340170405 8108379

[B8] Sonnberg S Fleming SB Mercer AA A truncated two-alpha-helix F-box present in poxvirus ankyrin-repeat proteins is sufficient for binding the SCF1 ubiquitin ligase complex J Gen Virol 2009 90 1224 1228 10.1099/vir.0.009324-0 19264588

[B9] Sonnberg S Seet BT Pawson T Fleming SB Mercer AA Poxvirus ankyrin repeat proteins are a unique class of F-box proteins that associate with cellular SCF1 ubiquitin ligase complexes Proc Natl Acad Sci USA 2008 105 10955 10960 10.1073/pnas.0802042105 18667692 PMC2504846

[B10] Skowyra D Craig KL Tyers M Elledge SJ Harper JW F-box proteins are receptors that recruit phosphorylated substrates to the SCF ubiquitin-ligase complex Cell 1997 91 209 219 10.1016/S0092-8674(00)80403-1 9346238

[B11] Hershko A Ciechanover A The ubiquitin system Annu Rev Biochem 1998 67 425 479 10.1146/annurev.biochem.67.1.425 9759494

[B12] Deshaies RJ SCF and Cullin/Ring H2-based ubiquitin ligases Annu Rev Cell Dev Biol 1999 15 435 467 10.1146/annurev.cellbio.15.1.435 10611969

[B13] Johnston JB Wang G Barrett JW Nazarian SH Colwill K Moran M McFadden G Myxoma virus M-T5 protects infected cells from the stress of cell cycle arrest through its interaction with host cell cullin-1 J Virol 2005 79 10750 10763 10.1128/JVI.79.16.10750-10763.2005 16051867 PMC1182661

[B14] Sperling KM Schwantes A Schnierle BS Sutter G The highly conserved orthopoxvirus 68k ankyrin-like protein is part of a cellular SCF ubiquitin ligase complex Virology 2008 374 234 239 10.1016/j.virol.2008.02.018 18353424

[B15] van Buuren N Couturier B Xiong Y Barry M Ectromelia virus encodes a novel family of F-box proteins that interact with the SCF complex J Virol 2008 82 9917 9927 10.1128/JVI.00953-08 18684824 PMC2566254

[B16] Maeda I Ohta T Koizumi H Fukuda M In vitro ubiquitination of cyclin D1 by ROC1-CUL1 and ROC1-CUL3 FEBS Lett 2001 494 181 185 10.1016/S0014-5793(01)02343-2 11311237

[B17] Werden SJ Lanchbury J Shattuck D Neff C Dufford M McFadden G The myxoma virus m-t5 ankyrin repeat host range protein is a novel adaptor that coordinately links the cellular signaling pathways mediated by Akt and Skp1 in virus-infected cells J Virol 2009 83 12068 12083 10.1128/JVI.00963-09 19776120 PMC2786711

[B18] Sperling KM Schwantes A Staib C Schnierle BS Sutter G The orthopoxvirus 68-kilodalton ankyrin-like protein is essential for DNA replication and complete gene expression of modified vaccinia virus Ankara in nonpermissive human and murine cells J Virol 2009 83 6029 6038 10.1128/JVI.01628-08 19357172 PMC2687370

[B19] Bradley RR Terajima M Vaccinia virus K1L protein mediates host-range function in RK-13 cells via ankyrin repeat and may interact with a cellular GTPase-activating protein Virus Res 2005 114 104 112 10.1016/j.virusres.2005.06.003 16039000

[B20] Shisler JL Jin XL The vaccinia virus K1L gene product inhibits host NF-kappaB activation by preventing IkappaBalpha degradation J Virol 2004 78 3553 3560 10.1128/JVI.78.7.3553-3560.2004 15016878 PMC371086

